# Enrollment of adolescents and young adults onto SWOG cancer research network clinical trials: A comparative analysis by treatment site and era

**DOI:** 10.1002/cam4.2891

**Published:** 2020-02-03

**Authors:** Michael E. Roth, Joseph M. Unger, Ann M. O'Mara, Mark A. Lewis, Troy Budd, Rebecca H. Johnson, Brad H. Pollock, Charles Blanke, David R. Freyer

**Affiliations:** ^1^ Division of Pediatrics The University of Texas MD Anderson Cancer Center Houston TX USA; ^2^ SWOG Cancer Research Network Statistics and Data Management Center Fred Hutchinson Cancer Research Center Seattle WA USA; ^3^ Community Oncology and Prevention Trials Research Group Division of Cancer Prevention National Cancer Institute Bethesda MD USA; ^4^ Intermountain Health Salt Lake City UT USA; ^5^ Department of Pediatric Hematology/Oncology Mary Bridge Children's Hospital and Health Center and Tacoma General Hospital Tacoma WA USA; ^6^ Department of Public Health Sciences and the UC Davis Comprehensive Cancer Center University of California Davis CA USA; ^7^ Southwest Oncology Group Chair's Office and Knight Cancer Center Institute Oregon Health & Science University Portland OR USA; ^8^ Departments of Pediatrics and Medicine Cancer and Blood Diseases Institute Children's Hospital Los Angeles USC Norris Comprehensive Cancer Center Keck School of Medicine University of Southern California Los Angeles CA USA

**Keywords:** adolescent and young adult, cancer, CCOP, clinical trials, enrollment, NCI, NCORP, SWOG

## Abstract

**Background:**

Few adolescents and young adults (AYAs, 15‐39 years old) enroll onto cancer clinical trials, which hinders research otherwise having the potential to improve outcomes in this unique population. Prior studies have reported that AYAs are more likely to receive cancer care in community settings. The National Cancer Institute (NCI) has led efforts to increase trial enrollment through its network of NCI‐designated cancer centers (NCICC) combined with community outreach through its Community Clinical Oncology Program (CCOP; replaced by the NCI Community Oncology Research Program in 2014).

**Methods:**

Using AYA proportional enrollment (the proportion of total enrollments who were AYAs) as the primary outcome, we examined enrollment of AYAs onto SWOG therapeutic trials at NCICC, CCOP, and non‐NCICC/non‐CCOP sites from 2004 to 2013 by type of site, study period (2004‐08 vs 2009‐13), and patient demographics.

**Results:**

Overall, AYA proportional enrollment was 10.1%. AYA proportional enrollment decreased between 2004‐2008 and 2009‐2013 (13.1% vs 8.5%, *P* < .001), and was higher at NCICCs than at CCOPs and non‐NCICC/non‐CCOPs (14.1% vs 8.3% and 9.2%, respectively; *P* < .001). AYA proportional enrollment declined significantly at all three site types. Proportional enrollment of AYAs who were Black or Hispanic was significantly higher at NCICCs compared with CCOPs or non‐NCICC/non‐CCOPs (11.5% vs 8.8, *P* = .048 and 11.5% vs 8.6%, *P* = .03, respectively).

**Conclusion:**

Not only did community sites enroll a lower proportion of AYAs onto cancer clinical trials, but AYA enrollment decreased in all study settings. Initiatives aimed at increasing AYA enrollment, particularly in the community setting with attention to minority status, are needed.

## Graphical abstract

From 2004‐2013, adolescents and young adults (AYA) proportional representation of AYAs on SWOG Cancer Research Network clinical trials decreased significantly across all treatment settings, including community‐based sites. Initiatives are needed to overcome barriers preventing limiting AYA trial enrollment.

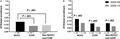

## 1 INTRODUCTION

Significant outcome disparities continue to plague adolescents and young adults (AYAs, 15‐39 years old) with cancer, particularly certain patient subsets defined by cancer type, presence of metastatic disease, and sociodemographic factors that include racial/ethnic minority and low socioeconomic status.^1^ Numerous studies have documented that enrollment of AYAs onto cancer clinical trials is significantly lower than that of younger patients and is similar to the low enrollment reported in older adults.^2^, ^3^, ^4^, ^5^, ^6^ Poor representation of AYAs on clinical trials limits improvements in survival for this population, including determination of optimal treatment approaches and age‐related differences in cancer and host biology.^2^, ^3^, ^4^, ^7^, ^8^, ^9^, ^10^, ^11^, ^12^

The US National Cancer Institute (NCI) has developed research networks focused on improving survival and health outcomes for cancer patients through the conduct of large‐scale clinical trials, including the National Clinical Trials Network (NCTN), the NCI Cancer Centers Program, and the NCI Community Oncology Research Program (NCORP). The NCTN provides funding and clinical trial infrastructure support to over 3000 sites via membership in NCTN network groups, including the SWOG Cancer Research Network (SWOG; formerly the Southwest Oncology Group). The NCI Cancer Centers Program consists of 70 NCI‐designated Cancer Centers (NCICC) that are members of the NCTN, including 49 Comprehensive Cancer Centers and 14 Clinical Cancer Centers. The Community Clinical Oncology Program (CCOP), predecessor to the NCORP, was launched in 1983 to increase access to NCI‐sponsored clinical trials by funding community‐based cancer treatment centers. It has accounted for one‐third of all NCI trial enrollments.^13^, ^14^, ^15^, ^16^, ^17^, ^18^ In 2014, the CCOP was transitioned to the NCORP, which retains the historical CCOP focus on clinical trial access in community settings.

Prior studies indicate that AYAs predominantly receive cancer care at community rather than urban academic sites, suggesting that community‐based sites with access to clinical trials should be well positioned to recruit this population.^19^, ^20^, ^21^, ^22^ However, a recent study of AYA enrollment onto Children's Oncology Group (COG) trials showed that, in fact, a significantly lower proportion of AYAs was enrolled at CCOP compared with non‐CCOP sites; further, proportional AYA enrollment at CCOP sites decreased significantly from 2004‐08 to 2009‐2013.^8^ To gain a more complete understanding of AYA enrollment patterns in both the pediatric and adult cooperative group settings, the objective of this current study was to evaluate AYA enrollment onto SWOG trials during the same time interval at NCI‐designated Cancer Centers, CCOP sites, and other sites that participate in NCI‐funded clinical trials. We hypothesized that the highest AYA proportional enrollment would be observed at CCOP sites and that, due to heightened awareness of AYA cancer disparities, AYA proportional enrollment would have increased at all sites over time.

## 2 METHODS

### 2.1 Data sources, definitions, and trial inclusion criteria

Enrollment data for all NCTN cooperative groups are regularly reported to the NCI. Utilizing the SWOG and NCI Division of Cancer Prevention (DCP) databases, we examined AYA proportional enrollment onto SWOG therapeutic trials, including Phase II and Phase III cancer treatment trials (n = 63) for selected cancers that were open during some portion of the period 2004‐2013. As previously described,^8^ AYA proportional enrollment was defined as the number of AYA patients (15‐39 years old) enrolled divided by the total number of all patients enrolled. Cancer treatment trials were defined as studies evaluating the impact of specific treatment regimens/approaches on survival outcomes. Trials for patients with both newly diagnosed and relapsed/refractory disease were included. Supportive care trials and nonintervention studies were excluded. Trials for cancer types that are rare among AYAs, eg, multiple myeloma and prostate cancer, were also excluded. Trials that met these inclusion criteria, along with those that were excluded, are listed in Supplemental Table S1.

### 2.2 Statistical analysis

We compared AYA proportional enrollment among (a) NCICC sites, (b) CCOP sites, and (c) non‐NCICC/non‐CCOP sites (ie, sites that were neither NCICC nor CCOP sites).^8^ Additionally, we evaluated temporal trends in enrollment patterns by era (2004‐2008 vs 2009‐2013), AYA age subsets (<30 vs ≥30 years) and race/ethnicity. Differences were assessed using Fisher's exact test and the *χ* ^2^ test of proportions.

## 3 RESULTS

### 3.1 Overall AYA clinical trial enrollment

During the overall period 2004‐2013, there were 23 328 total enrollments onto SWOG trials, of which 2380 were AYAs (proportional AYA enrollment = 10.2%). Total enrollments at NCICC sites, CCOP sites, and non‐NCICC/CCOP sites were 5604, 8856, and 13 868, respectively. Between 2004‐2008 and 2009‐2013, the number of open therapeutic trials increased from 34 to 47. Although the absolute number of AYAs enrolled increased slightly from 1130 to 1250 (by 120; +10.6%), the number of older patients increased to a much greater extent, from 7492 to 13 456 (by 5964; +55.7%). The proportion of AYAs enrolled thus declined markedly from 13.1% (1130/8622) to 8.5% (1250/14706; *P* < .001). Because AYA enrollment may be influenced by which trials are open at any given time, we determined proportional AYA enrollment only for those trials open during both study periods (n = 18) (Table S1) and found it also declined significantly from 13.7% to 10.5% (*P* < .001).

### 3.2 AYA clinical trial enrollment by type of treatment site

**Figure 1:**
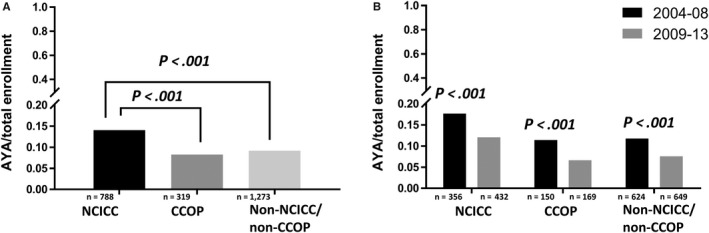
Proportion of therapeutic study enrollments that were adolescents and young adult (AYA) by treatment site type and time interval. AYA proportional enrollment was significantly greater at NCICC compared with CCOP and non‐NCICC/CCOPs during the years 2004‐2013 (Panel A). AYA proportional enrollment decreased significantly between the years 2004‐08 and 2009‐13 at NCICC, CCOP, and non‐NCICC/CCOP sites (Panel B). On both panels, n represents the number of enrollments

For the entire period 2004‐2013, NCICC sites demonstrated significantly higher proportional enrollment of AYAs compared with CCOP sites and non‐NCICC/CCOP sites (14.1% vs 8.3% and 9.2%, respectively; *P* < .001 for both comparisons), while the difference in proportional AYA enrollment between CCOP and non‐NCICC/non‐CCOP sites was not significant (*P* = .09; Figure 1A). Between the periods 2004‐2008 and 2009‐2013, AYA proportional enrollment decreased significantly at all three categories of sites (*P* < .001; Figure 1B). CCOPs experienced the largest decrease in proportional enrollment of AYAs followed by non‐NCICC/CCOPs and NCICCs (−42.1%, −35.7%, and −31.9%, respectively).

### 3.3 AYA clinical trial enrollment by age

**Figure 2:**
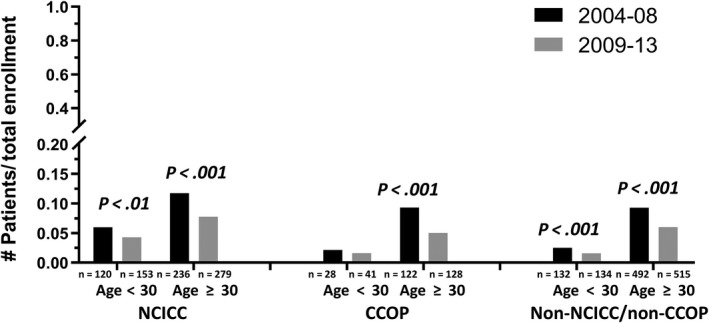
Proportion of therapeutic study enrollments that were adolescents and young adult (AYA) by age group, treatment site type, and time interval. The figure shows that the proportional enrollment for patients 15‐29 y old was low at all sites. Between 2004‐2008 and 2009‐2013, for patients 30‐39 y old proportional enrollment decreased significantly at all sites. For patients 15‐29 y old, proportional enrollment decreased significantly at NCICCs and non‐NCICC/non‐CCOPs, but not CCOPs. The value for n represents the number of enrollments

In order to characterize the impact of age within the broad range of AYAs, we compared proportional enrollment for patients 15‐29 vs 30‐39 years old. The group with 15‐29 year‐olds had a significantly lower proportion of enrollment than those aged 30‐39 at NCICC sites (4.9% [n = 273] vs 9.2% [n = 515], *P* < .001); at CCOP sites (1.8% [n = 69] vs 6.5% [n = 250], *P* < .001); and at non‐NCICC/non‐CCOP sites (1.9% [n = 266] vs 7.3% [n = 1007], *P* < .001). Enrollment for both these AYA subgroups significantly decreased over time at NCICCs and non‐NCICC/non‐CCOPs, and proportional enrollment for patients age 30‐39 significantly decreased at CCOPs (Figure 2).

### 3.4 AYA clinical trial enrollment by race and ethnicity

**Figure 3:**
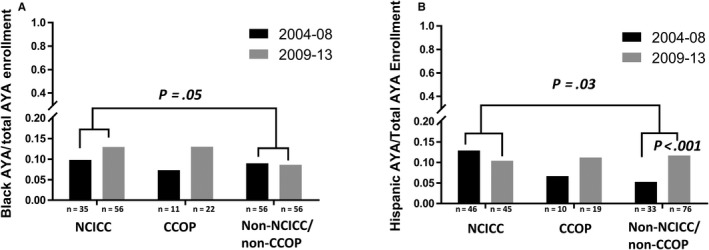
Proportion of adolescents and young adult (AYA) therapeutic study enrollments that were Black or Hispanic by treatment site type and time interval. The proportion of AYA enrollments who were Black was significantly higher at NCICCs compared with non‐NCICC/CCOPs (Panel A). The proportion of AYA enrollments who were Hispanic was significantly higher at NCICCs compared with non‐NCICC/non‐CCOPs (Panel B). The proportion of AYA enrollments who were Hispanic increased significantly only at non‐NCICC/non‐CCOP sites between 2004‐08 and 2009‐2013. On both panels, n represents the number of enrollments

Because few studies have assessed minority AYA enrollment onto cancer clinical trials, we also evaluated AYA enrollment by race and ethnicity. At NCICC, CCOP, and non‐NCICC/non‐CCOP sites the proportions of AYA enrollments that were Black was 11.5% (n = 91), 10.3% (n = 33), and 8.8% (n = 112), respectively; the proportions that were Hispanic were 11.5% (n = 91), 9.1% (n = 29), and 8.6% (n = 109); the proportions that were Asian/Pacific Islander were 6.2% (n = 49), 2.2% (n = 7), and 6.7% (n = 85); and the proportions that were Native American were 0.6% (n = 5), 1.3% (n = 4), and 0.7% (n = 7), respectively. AYA enrollment for the two largest AYA minority groups, Black and Hispanic patients, is shown in Figure 3. Both the proportional enrollment of AYAs who were Black and of those who were Hispanic were significantly higher at NCICCs compared with non‐NCICC/non‐CCOPs (*P* = .05 and *P* = .03, respectively; Figure 3A,B). Also, the proportional enrollment of AYAs who were Asian/Pacific Islander was significantly higher at NCICCs compared to CCOPs (*P* = .009). The proportion of Hispanic AYAs increased significantly over time only at non‐NCICC/non‐CCOP sites (8.6% vs 11.7%, *P* < .001; Figure 3B). There was no statistically significant change in Black, Asian/Pacific Islander, or Native American AYA enrollment in any site type (Figure 3A). However, the numbers of AYA patients in these groups were particularly small.

## 4 DISCUSSION

While the overall NCI clinical trial enterprise has been highly successful,^13^, ^17^ our studies of AYA enrollment in two cooperative oncology groups document that, among all categories of institutions offering NCI‐funded trials, AYA representation as a proportion of total enrollments is lowest, and decreased the most over time, at community‐based sites. This finding is both unexpected and worrisome, given that AYAs predominantly receive their cancer care in the community setting.^8^, ^19^, ^20^, ^22^ This study of SWOG AYA enrollment, combined with our previous study of COG AYA enrollment^8^ demonstrates that recruitment of AYAs onto NCI‐sponsored clinical trials is a considerable challenge for CCOP and non‐NCICC/non‐CCOP sites. Despite numerous publications documenting low levels of AYA participation in cancer clinical trials,^4^, ^6^, ^7^, ^11^ as well as those highlighting AYA cancer outcomes disparities that might be ameliorated through the conduct of cancer clinical trials,^4^, ^7^, ^8^ our two studies reveal that proportional representation of AYAs on NCI‐funded clinical trials actually fell significantly between 2004 and 2013. This study makes clear that simply providing community sites with access to NCI‐sponsored clinical trials is not enough to recruit a substantial proportion of AYAs. Our results add urgency to the well‐documented need to increase AYA enrollment, which, despite heightened awareness, is getting worse as reflected by this measure. Our findings suggest that offering NCI‐funded trials in the community setting must be accompanied by targeted recruitment strategies aimed at the AYA population in order to be as successful at AYA participation as large academic sites.

Maximizing AYA enrollment onto clinical trials is important because AYAs constitute a relatively small number of participants on either pediatric or adult studies. For example, on AAML0531, a COG study for patients with acute myeloid leukemia (AML) age 1 month‐29.99 years, of 1070 participants only 163 (15%) were over 15 years of age, and only 13 (1%) were 21 years or older.^23^ Conversely, on S0106, a SWOG trial for patients with AML age 18‐60 years, of 595 participants only 57 (19%) were less than age 35 years.^24^ With AYAs being outnumbered in this fashion, it is extremely difficult to determine AYA‐specific outcomes or to design studies with AYA‐specific aims. Even if NCTN groups were to combine efforts and develop AYA‐specific trials, for some cancer types the number of participants may still be marginal for achieving adequate power to detect outcome differences in treatment comparisons. Currently, S1826 is an NCTN trial for treatment of advanced‐stage Hodgkin lymphoma jointly developed by SWOG and COG for patients age 12 years and older, thus encompassing the entire AYA age range (NCT03907488). For appropriate cancers, such collaborative efforts probably represent the best mechanism for studying AYA‐specific outcomes. Although it is generally acknowledged that the chief value of cancer clinical trials is to improve outcomes over time for patient populations, one study found that AYAs were nearly three times more likely to receive appropriate initial therapy if they were enrolled on a clinical trial than if not enrolled.^25^ Thus, for AYAs an additional incentive for clinical trial participation may be optimizing treatment for individual patients.

Our prior study that demonstrated low and falling AYA proportional enrollment onto COG trials during 2008‐2014 raised the question whether AYA enrollment onto adult cooperative group trials may have increased concurrently and accounted for that drop.^8^ This study, conducted for the same time period, clearly shows that AYA proportional enrollment onto adult cooperative group trials also decreased over time. Due to the AYA age range encompassing both older pediatric and younger adult patients, effective strategies for increasing AYA enrollment require close collaboration and cooperation between COG and the adult focused NCTN groups, as well as between pediatric and medical oncology providers at the site level.

Our results demonstrate that AYAs in their twenties represent a very small proportion of patients on adult‐focused cancer clinical trials. The very low enrollment proportion of younger AYAs (2.6%) compared with older AYAs (7.6%) in our study likely reflects, at least in part, the lower incidence of cancer among AYAs. To better understand patterns of AYA enrollment by type of institution, we would need to know the total number of incident cancer cases diagnosed at the treatment centers included in this study. Unfortunately, reporting these denominator data to NCI is not currently required. In one study of AYA clinical trial enrollment, cancer registry data were used to identify incident cases of AYA cancer that were then individually linked to clinical trial enrollment records [10], but such linkage ability is not uniformly available. This inability to determine accurately the “AYA denominator” of new cancer cases diagnosed at treatment sites for any given reporting period remains a major roadblock to acquiring a fully informed understanding of AYA clinical trial enrollment patterns. Further, the absolute number of younger AYAs is very small (only 608 in this age group were enrolled onto these trials during this period), making it difficult to conduct studies for young AYAs with specific cancer types. Such trials are necessary for defining optimal cancer treatment including the role of new agents, effective supportive care approaches, and elucidating the underlying prognostic significance of differences in cancer and host biology that can lead to improved AYA outcomes. The success of such trials will necessarily involve collaboration between the adult NCTN groups and the COG.

Our study suggests that AYA racial and ethnic minorities were underrepresented on the clinical trials examined here. We found that 9.9% of AYAs enrolled were Black and 9.6% were Hispanic. While our study methodology precludes knowing how these precentages relate to the actual numbers of minority AYAs diagnosed with cancer, the proportions we observed are well below US population percentages for these groups, which are in the range of approximately 17% and 14%, respectively.^26^ Few studies have examined the relationship between race/ethnicity and cancer trial enrollment for AYA patients. In a case‐linked analysis of AYA clinical trial enrollment in the context of an NCI‐designated comprehensive cancer center, Latinos were found to be more likely to enroll than non‐Hispanic whites, while Blacks were less likely.^3^ Understanding the impact of sociodemographic variables on AYA clinical trial enrollment is increasingly relevant with minorities projected to increase from 36% of the total US population in 2010 to approximately 50% in 2040. As in the case of age, our study calls attention to the importance of being able to determine accurately the “AYA denominator” in all categories of race, ethnicity and SES groups. Our comparisons of minority enrollment showed small or nonsignificant changes over time, likely due to limited numbers of AYA patients in these groups.

Strengths of our study include the large number of enrollments analyzed (n = 23 328), the use of well‐established and robust data sources (the SWOG and NCI DCP databases), the assessment of enrollment patterns over a recent decade that aligns with our previous study of AYA enrollments onto COG trials, and the gaining of new insights into Black and Hispanic AYA enrollment. Limitations include the methodology of utilizing AYA proportional enrollment (proportion of study enrollments that are AYA) in lieu of the proportion of enrolled AYAs among all AYAs diagnosed with cancer at each site. As discussed earlier, the inability to obtain the “AYA denominator,” that is, the total number of AYAs diagnosed with cancer at each site, precludes that type of assessment. While overall study enrollments increased by 71% (6084 enrollments) between the two study periods (largely resulting from the increase in the total number of open trials), AYA enrollments accounted for only 2% (120 enrollments) of this increase. In this regard, it is important to emphasize that trials not relevant to AYAs (eg, prostate) were excluded from our study.

In 2014 the CCOP transitioned into the NCORP to expand the reach and impact of the program and align its goals with the NCI National Clinical Trials Network.^27^, ^28^ Given the limited and decreasing AYA proportional enrollment on NCTN cooperative group trials at these community sites, a study led by the COG AYA Oncology Discipline Committee is in progress to identify the most prevalent and targetable AYA enrollment barriers at NCORP sites. Having documented that AYA representation on SWOG Cancer Research Network clinical trials decreased over the past decade, it is hoped that these results will stimulate additional research to develop and implement interventions aimed at increasing AYA enrollment, most notably at community sites, onto NCI‐sponsored cancer clinical trials.

## CONFLICT OF INTEREST

The authors do not have any conflict of interest to disclose.

## AUTHOR CONTRIBUTIONS

Michael Roth and David Freyer participated in the development of the study concept, performed data analysis and interpretation of the data and participated in the writing of the manuscript. Joseph Unger participated in the development of the study concept, provided data for the study, performed data analysis and interpretation of the data and participated in the writing of the manuscript. Troy Budd provided data for the study. Ann O'Mara, Mark Lewis, Rebecca Johnson and Brad Pollock participated in the development of the study concept, interpretation of the data and in the writing of the manuscript. Charles Blanke participated in the interpretation of the data, and in the writing of the manuscript.

## Data Availability

Data available on request from the authors.
