# Supplementary material for: Enrollment of adolescents and young adults onto SWOG cancer research network clinical trials: A comparative analysis by treatment site and era
Source: Cancer Med. 2020 Feb 3;9(6):2146–52. doi: 10.1002/cam4.2891 (PMC7064039; doi:10.1002/cam4.2891)
Supplement: Supplementary file 1 [file CAM4-9-2146-s001.docx]

**Supplemental Table 1.**

| **Study Number** | **Trial Title** | **Study Era** | **Included/ Excluded** | **Total Accrual** | **Trial Type** |
| --- | --- | --- | --- | --- | --- |
| S0001 | A Phase III Study of Radiation Therapy (RT) and 06-Benzylguanine (O6-BG) Plus BCNU Versus RT and BCNU Alone for Newly Diagnosed Glioblastoma Multiforme (GBM) and Gliosarcoma | 2004-08 | Included | 183 | Newly diagnosed |
| S0008 | Phase III Trial of High Dose Interferon Alpha-2b Versus Cisplatin, Vinblastine, DTIC Plus IL-2 and Interferon in Patients with High Risk Melanoma | 2004-08 | Included | 432 | Newly diagnosed/ Relapsed |
| S0012 | A Comparative Randomized Study of Standard Doxorubicin and Cyclophosphamide Followed by Weekly Paclitaxel vs. Weekly Doxorubicin and Daily Oral Cyclophosphamide Plus G-CSF Followed by Weekly Paclitaxel as Neoadjuvant Therapy for Inflammatory and Locally Advanced Breast Cancer | 2004-08 | Included | 399 | Newly diagnosed |
| S0016 | A Phase III Trial of CHOP + Rituximab vs CHOP + Iodine-131-Labeled Monoclonal Anti-B1 Antibody (Tositumomab) for Treatment of Newly Diagnosed Follicular Non-Hodgkin's Lymphomas | 2004-08 | Included | 571 | Newly diagnosed |
| S0106 | A Phase III Study of the Addition of Gemtuzumab Ozogamicin (Mylotarg) During Induction Therapy Versus Standard Induction with Daunomycin and Cytosine Arabinoside Followed by Consolidation and Subsequent Randomization to Post-Consolidation Therapy with Gemtuzumab Ozogamicin (Mylotarg) or No Additional Therapy for Patients Under Age 61 with Previously Untreated De Novo Acute Myeloid Leukemia (AML) | 2004-08 | Included | 637 | Newly diagnosed |
| S0117 | A Phase II Study of Gemtuzumab Ozogamicin (Mylotarg) and Standard Dose Ara-C for Patients with Relapsed Acute Myeloid Leukemia (AML) | 2004-08 | Included | 33 | Relapsed |
| S0200 | A Phase III Randomized Study of Pegylated Liposomal Doxorubicin Plus Carboplatin Versus Carboplatin in Platinum-Sensitive Patients with Recurrent Epithelial Ovarian or Peritoneal Carcinoma After Failure of Initial Platinum-Based Chemotherapy | 2004-08 | Included | 61 | Relapsed |
| S0213 | Pilot Trial of Hyper-CVAD and Methotrexate/Ara C + Rituximab in Patients with Previously Untreated Mantle Cell Lymphoma | 2004-08 | Included | 56 | Newly diagnosed |
| S0215 | Docetaxel and Vinorelbine Plus Filgrastim with Weekly Trastuzumab for HER-2 Positive, Stage IV Breast Cancer | 2004-08 | Included | 76 | Newly diagnosed/ Relapsed |
| S0220 | A Phase II Trial of Induction Chemoradiotherapy with Cisplatin/Etoposide Followed by Surgical Resection, Followed by Docetaxel, for Non-Small Cell Lung Cancer Involving the Superior Sulcus (Pancoast Tumors) | 2004-08 | Included | 46 | Newly diagnosed |
| S0313 | Evaluation of CHOP Plus Involved Field Radiotherapy Followed by Yttrium-90 Ibritumomab Tiuxetan for Stages I, IE, and Non-Bulky Stages II and IIE, CD20 Positive, High-Risk Localized, Aggressive Histologies of Non-Hodgkin's Lymphoma, Phase II | 2004-08 | Included | 46 | Newly diagnosed |
| S0356 | Oxaliplatin Plus Protracted Infusion 5-Fluorouracil and Radiation for Potentially Curable Esophageal Cancer: A Phase II Trial with Molecular Correlates | 2004-08 | Included | 98 | Newly diagnosed |
| S0526 | Phase II Trial of Pemetrexed in Patients with Selected Stage IIIB and IV Bronchioloalveolar Carcinoma (BAC) | 2004-08 | Included | 27 | Newly diagnosed/ Relapsed |
| S0601 | A Phase II Study of Combination Rituximab-CHOP and Bortezomib (Velcade) (R-CHOP-V) Induction Therapy Followed by Bortezomib Maintenance (VM) Therapy for Patients with Newly Diagnosed Mantle Cell Lymphoma | 2004-08 | Included | 68 | Newly diagnosed |
| S9704 | A Randomized Phase III Trial Comparing Early High Dose Chemoradiotherapy and an Autologous Stem Cell Transplant to Conventional Dose CHOP Chemotherapy Plus Rituximab for CD20 + B Cell Lymphomas (with Possible Late Autologous Stem Cell Transplant) for Patients with Diffuse Aggressive Non-Hodgkin's Lymphoma in the High-Intermediate and High Risk International Classification Prognostic Groups | 2004-08 | Included | 419 | Newly diagnosed |
| S9430 | A Phase II Trial of Complete Surgical Resection for Stage IV Melanoma - Surgical Resection with Biological Evaluation and Clinical Follow-Up | 2004-08 | Included | 77 | Newly diagnosed/ Relapsed |
| S0927 | A Randomized Placebo-Controlled Trial of Omega-3-Fatty Acid for the Control of Aromatase Inhibitor-Induced Musculoskeletal Pain and Stiffness in Women with Early Stage Breast Cancer, Phase III | 2009-13 | Excluded | 262 | Cancer Control |
| S0715 | Randomized Placebo-Controlled Trial Of Acetyl-L-Carnitine (ALC) For the Prevention of Taxane Induced Neuropathy Phase III | 2009-13 | Excluded | 437 | Cancer Control |
| S0812 | A Randomized Double-Blind Placebo-Controlled Biomarker Modulation Study of High Dose Vitamin D in Premenopausal Women at High-Risk for Breast Cancer Phase IIB | 2009-13 | Excluded | 208 | Cancer Control |
| S0820 | A Double Blind Placebo-Controlled Trial of Eflornithine and Sulindac to Prevent Recurrence of High Risk Adenomas and Second Primary Colorectal Cancers in Patients with Stage 0-III Colon or Rectal Cancer, Phase III- Preventing Adenomas of the Colon with Eflornithine and Sulindac (PACES) | 2009-13 | Excluded | 104 | Cancer Control |
| S1304 | A Phase II Randomized Study Comparing Two Doses of Carfilzomib (NSC-756640) with Dexamethasone for Multiple Myeloma Patients with Relapsed or Refractory Disease | 2009-13 | Excluded | 143 | Relapsed |
| S1011 | A Phase III Surgical Trial to Evaluate the Benefit of a Standard Versus an Extended Pelvic Lymphadenectomy Performed at Time of Radical Cystectomy for Muscle Invasive Urothelial Cancer | 2009-13 | Excluded | 627 | Newly diagnosed |
| S1008 | Feasibility Study of a Physical Activity and Dietary Change Weight Loss Intervention in Breast and Colorectal Cancer Survivors, Phase II | 2009-13 | Excluded | 50 | Cancer Control |
| S1200 | Randomized Blinded Sham-and Waitlist-Controlled Trial of Acupuncture for Joint Symptoms Related to Aromatase Inhibitors in Women With Early Stage Breast Cancer | 2009-13 | Excluded | 212 | Cancer Control |
| S1202 | A Randomized Placebo-Controlled Phase III Study of Duloxetine for Treatment of Aromatase Inhibitor (AI)-Associated Musculoskeletal Symptoms in Women with Early Stage Breast Cancer | 2009-13 | Excluded | 299 | Cancer Control |
| S0709 | A Phase II Selection Design of Pharmacodynamic Separation of Carboplatin/Paclitaxel/OSI-774 (Erlotinib; NSC-718781) or OSI-774 Alone in Advanced Non-Small Cell Lung Cancer (NSCLC) Patients with Performance Status 2 (PS-2) Selected by Serum Proteomics | 2009-13 | Included | 59 | Newly diagnosed/ Relapsed |
| S0713 | A Phase II Study of Oxaliplatin, Capecitabine, Cetuximab and Radiation in Pre-Operative Therapy of Rectal Cancer | 2009-13 | Included | 83 | Newly diagnosed |
| S0720 | Phase II ERCC1 and RRM1-Based Adjuvant Therapy Trial in Patients with Stage I Non-Small Cell Lung Cancer (NSCLC) | 2009-13 | Included | 85 | Newly diagnosed |
| S0722 | A Phase II Trial of mTOR Inhibitor, Everolimus (RAD001), in Malignant Pleural Mesothelioma (MPM) | 2009-13 | Included | 61 | Newly diagnosed/ Relapsed |
| S0800 | A Randomized Phase II Trial of Weekly Nanoparticle Albumin Bound Paclitaxel (Nab-Paclitaxel) (NSC-736631) with or Without Bevacizumab, Either Preceded by or Followed by Q 2 Week Doxorubicin (A) and Cyclophosphamide (C) Plus Pegfilgrastim (PEG-G) as Neoadjuvant Therapy for Inflammatory and Locally Advanced HER-2/Neu Negative Breast Cancer | 2009-13 | Included | 215 | Newly diagnosed |
| S0801 | A Phase II Study of Iodine-131-Labeled Tositumomab in Combination with Cyclophosphamide, Doxorubicin, Vincristine, Prednisone and Rituximab Therapy for Patients with Advanced Stage Follicular Non-Hodgkin's Lymphoma | 2009-13 | Included | 87 | Newly diagnosed |
| S0802 | A Randomized Phase II Trial of Weekly Topotecan with and without AVE0005 (Aflibercept; NSC-724770) in Patients with Platinum Treated Extensive Stage Small Cell Lung Cancer (E-SCLC) | 2009-13 | Included | 192 | Relapsed |
| S0809 | A Phase II Trial of Adjuvant Capecitabine/Gemcitabine Chemotherapy Followed by Concurrent Capecitabine and Radiotherapy in Extrahepatic Cholangiocarcinoma (EHCC) | 2009-13 | Included | 105 | Newly diagnosed |
| S0816 | A Phase II Trial of Response-Adapted Therapy of Stage III-IV Hodgkin Lymphoma Using Early Interim FDG-PET Imaging | 2009-13 | Included | 371 | Newly diagnosed |
| S0819 | A Randomized, Phase III Study Comparing Carboplatin/Paclitaxel or Carboplatin/Paclitaxel/Bevacizumab with or Without Concurrent Cetuximab in Patients with Advanced Non-Small Cell Lung Cancer (NSCLC) | 2009-13 | Included | 1333 | Newly diagnosed/ Relapsed |
| S0826 | A Phase II Trial of SCH 727965 (NSC 747135) in Patients with Stage IV Melanoma | 2009-13 | Included | 72 | Newly diagnosed/ Relapsed |
| S0904 | Randomized Phase II Study of Docetaxel Followed by Vandetanib (ZD6474) vs. Docetaxel Plus Vandetanib in Patients with Persistent or Recurrent Epithelial Ovarian, Fallopian Tube, or Primary Peritoneal Carcinoma | 2009-13 | Included | 131 | Relapsed |
| S0910 | A Phase II Study of Epratuzumab (NSC-716711) in Combination with Cytarabine and Clofarabine for Patients with Relapsed or Refractory Ph-Negative Precursor B-Cell Acute Lymphoblastic Leukemia (ALL) | 2009-13 | Included | 35 | Relapsed |
| S0916 | A Phase II, Window Trial of the Anti-CCR2 Antibody MLN1202 (NSC-751448) in Patients with Bone Metastases | 2009-13 | Included | 44 | Newly diagnosed/ Relapsed |
| S0919 | A Phase II Study of Idarubicin and Ara-C in Combination with Pravastatin for Poor-Risk Acute Myelogenous Leukemia (AML) | 2009-13 | Included | 109 | Relapsed |
| S0931 | EVEREST: EVErolimus for Renal Cancer Ensuing Surgical Therapy, A Phase III Study | 2009-13 | Included | 1537 | Newly diagnosed |
| S0933 | Phase II Study of RO4929097 (NSC-749225) in Advanced Melanoma | 2009-13 | Included | 36 | Newly diagnosed |
| S0941 | Phase II Study of Sorafenib (NSC-724772) and Erlotinib (NSC-718781) in Patients with Advanced Gallbladder Carcinoma or Cholangiocarcinoma | 2009-13 | Included | 40 | Newly diagnosed/ Relapsed |
| S1001 | A Phase II Trial of PET-Directed Therapy for Limited Stage Diffuse Large B-Cell Lymphoma (DLBCL) | 2009-13 | Included | 158 | Newly diagnosed |
| S1005 | A Phase II Study of MK-2206 (NSC-749607) as Second Line Therapy for Advanced Gastric and Gastroesophageal Junction Cancer | 2009-13 | Included | 75 | Relapsed |
| S1007 | A Phase III, Randomized Clinical Trial of Standard Adjuvant Endocrine Therapy +/- Chemotherapy in Patients with 1-3 Positive Nodes, Hormone Receptor-Positive and HER2-Negative Breast Cancer with Recurrence Score (RS) of 25 or Less. RxPONDER: A Clinical Trial Rx for Positive Node, Endocrine Responsive Breast Cancer | 2009-13 | Included | 7874 | Newly diagnosed |
| S1106 | A Randomized Phase II Trial of R-HCVAD/MTX/ARA-C Induction Followed by Consolidation with an Autologous Stem Cell Transplant vs. R-Bendamustine Induction Followed by Consolidation with an Autologous Stem Cell Transplant for Patients </= 65 Years of Age with Previously Untreated Mantle Cell Lymphoma | 2009-13 | Included | 53 | Newly diagnosed |
| S1107 | Parallel (Randomized) Phase II Evaluation of ARQ 197 and ARQ 197 in Combination with Erlotinib in Papillary Renal Cell Carcinoma | 2009-13 | Included | 55 | Newly diagnosed/ Relapsed |
| S1108 | Phase II Trial of the Aurora Kinase A Inhibitor MLN8237, in Relapsed or Refractory Peripheral T-Cell Non-Hodgkin Lymphoma | 2009-13 | Included | 42 | Relapsed |
| S1115 | Randomized Phase II Clinical Trial of AZD6244 Hydrogen Sulfate (NSC-748727) and MK-2206 (NSC-749607) Vs MFOLFOX in Patients with Metastatic Pancreatic Cancer After Prior Chemotherapy | 2009-13 | Included | 137 | Relapsed |
| S1117 | A Randomized Phase II/III Study of Azacitidine in Combination with Lenalidomide (NSC-703813) vs. Azacitidine Alone vs. Azacitidine in Combination with Vorinostat (NSC-701852) for Higher-Risk Myelodysplastic Syndromes (MDS) and Chronic Myelomonocytic Leukemia (CMML) | 2009-13 | Included | 282 | Newly diagnosed |
| S1201 | A Randomized Phase II Pilot Study Prospectively Evaluating Treatment for Patients Based on ERCC1(Excision Repair Cross-Complementing 1) for Advanced/Metastatic Esophageal, Gastric or Gastroesophageal Junction (GEJ) Cancer | 2009-13 | Included | 264 | Newly diagnosed/ Relapsed |
| S1203 | A Randomized Phase III Study of Standard Cytarabine Plus Daunorubicin (7+3) Therapy or Idarubicin with High Dose Cytarabine (IA) Versus IA with Vorinostat (NSC-701852) (IA+V) in Younger Patients with Previously Untreated Acute Myeloid Leukemia (AML) | 2009-13 | Included | 755 | Newly diagnosed/ Relapsed |
| S1207 | Phase III Randomized, Placebo-Controlled Clinical Trial Evaluating the Use of Adjuvant Endocrine Therapy +/- One Year of Everolimus in Patients with High-Risk, Hormone Receptor-Positive and HER2/Neu Negative Breast Cancer. e^3 Breast Cancer Study-Evaluating Everolimus with Endocrine Therapy. | 2009-13 | Included | 973 | Newly diagnosed |
| S0605 | A Phase II Study of Lenalidomide (Revlimid, NSC-703813) for Previously Untreated Non-M3, Deletion 5q Acute Myeloid Leukemia (AML) in Patients Age 60 or Older Who Decline Remission Induction Chemotherapy | Both | Excluded | 41 | Newly diagnosed |
| S0703 | A Phase II Trial of Azacitidine (NSC-102816) Plus Gemtuzumab Ozogamicin (NSC-720568) as Induction and Post-Remission Therapy in Patients of Age 60 and Older with Previously Untreated Non-M3 Acute Myeloid Leukemia | Both | Excluded | 142 | Newly diagnosed |
| S0337 | A Phase III Blinded Study of Immediate Post-TURBT Instillation of Gemcitabine Versus Saline in Patients with Newly Diagnosed or Occasionally Recurring Grade I/II Superficial Bladder Cancer | Both | Excluded | 416 | Newly diagnosed |
| S0353 | Phase II Study of Intravesical Gemcitabine in Patients with Superficial Bladder Cancer Who Have Progressed Despite Intravesical BCG | Both | Excluded | 58 | Relapsed |
| S0777 | A Randomized Phase III Trial of CC-5013 (Lenalidomide, NSC-703813) and Low Dose Dexamethasone (LLD) Versus Bortezomib (PS-341, NSC-681239), Lenalidomide and Low Dose Dexamethasone (BLLD) for Induction, in Patients with Previously Untreated Multiple Myeloma Without an Intent for Immediate Autologous Stem Cell Transplant | Both | Excluded | 525 | Newly diagnosed |
| S0000A | Prevention of Alzheimer's Disease with Vitamin E and Selenium (PREADVISE) Phase III Ancillary to S0000 SELECT | Both | Excluded | 7553 | Cancer Control |
| S0000D | A Study of the Effect of Vitamin E and/or Selenium on Adenomatous Colorectal Polyps (ACP) in Participants Enrolled in SELECT, Phase II Ancillary to S0000-SELECT | Both | Excluded | 8097 | Cancer Control |
| S0230 | Phase III Trial of LHRH Analog Administration During Chemotherapy to Reduce Ovarian Failure Following Chemotherapy in Early Stage, Hormone-Receptor Negative Breast Cancer | Both | Excluded | 257 | Cancer Control |
| S9630 | A Randomized Comparison of Medroxyprogesterone Acetate (MPA) and Observation for Prevention of Endometrial Pathology in Postmenopausal Breast Cancer Patients Treated with Tamoxifen | Both | Excluded | 313 | Cancer Control |
| S0029 | Single Agent Docetaxel for Metastatic Breast Cancer in Patients Aged 70 Years and Older (And in a Cohort of Patients Younger than 60 Years) | Both | Included | 27 | Newly diagnosed |
| S0221 | Phase III Trial of Continuous Schedule AC + G vs. Q 2 Week Schedule AC, Followed by Paclitaxel Given Either Every 2 Weeks or Weekly for 12 Weeks as Post-Operative Adjuvant Therapy in Node-Positive or High-Risk Node-Negative Breast Cancer | Both | Included | 3294 | Newly diagnosed |
| S0226 | Phase III Randomized Trial of Anastrozole Versus Anastrozole and Fulvestrant as First Line Therapy for Post Menopausal Women with Metastatic Breast Cancer | Both | Included | 707 | Newly diagnosed |
| S0307 | Phase III Trial of Bisphosphonates as Adjuvant Therapy for Primary Breast Cancer | Both | Included | 6097 | Newly diagnosed |
| S0325 | A Phase IIb Study of Molecular Responses to Imatinib, at Standard or Increased Doses, or Dasatinib (BMS-354825) (NSC-732517) for Previously Untreated Patients with Chronic Myelogenous Leukemia (CML) in Chronic Phase | Both | Included | 403 | Newly diagnosed |
| S0350 | Phase II Trial of Cisplatin Plus Etoposide Plus Gemcitabine Plus Solumedrol (PEGS) in Peripheral T-Cell Non-Hodgkin's Lymphoma | Both | Included | 34 | Newly diagnosed/ Relapsed |
| S0410 | Tandem Autologous Stem Cell Transplantation for Patients with Primary Progressive or Recurrent Hodgkin's Disease (A BMT Study), Phase II | Both | Included | 98 | Relapsed |
| S0433 | Iodine-131-Labeled Monoclonal Anti-B1 Antibody (I-131Tositumomab) in Combination with Cyclophosphamide, Doxorubicin, Vincristine, Prednisone and Rituximab Therapy for Patients with Advanced Stage Diffuse Large B-Cell NHL: A Phase II Study | Both | Included | 86 | Newly diagnosed |
| S0500 | A Randomized Phase III Trial to Test the Strategy of Changing Therapy Versus Maintaining Therapy for Metastatic Breast Cancer Patients Who Have Elevated Circulating Tumor Cell Levels at First Follow-Up Assessment | Both | Included | 624 | Newly diagnosed/ Relapsed |
| S0502 | A Phase III Randomized Study of Imatinib, with or Without Bevacizumab (NSC-704865), in Patients with Metastatic or Unresectable Gastrointestinal Stromal Tumors | Both | Included | 12 | Newly diagnosed/ Relapsed |
| S0518 | Phase III Prospective Randomized Comparison of Depot Octreotide Plus Interferon Alpha Versus Depot Octreotide Plus Bevacizumab (NSC #704865) in Advanced, Poor Prognosis Carcinoid Patients | Both | Included | 427 | Newly diagnosed/ Relapsed |
| S0521 | A Randomized Trial of Maintenance Versus Observation for Patients with Previously Untreated Low and Intermediate Risk Acute Promyelocytic Leukemia (APL), Phase III | Both | Included | 105 | Newly diagnosed |
| S0533 | A Pilot Trial of Cisplatin/Etoposide/Radiotherapy Followed by Consolidation Docetaxel and the Addition of Bevacizumab (NSC-704865) in Three Cohorts of Patients with Inoperable Locally Advanced Stage III Non-Small Cell Lung Cancer | Both | Included | 28 | Newly diagnosed |
| S0535 | A Phase II Study of ATRA, Arsenic Trioxide and Gemtuzumab Ozogamicin in Patients with Previously Untreated High-Risk Acute Promyelocytic Leukemia | Both | Included | 78 | Newly diagnosed |
| S0600 | Phase III Trial of Irinotecan-Based Chemotherapy Plus Cetuximab (NSC-714692) or Bevacizumab (NSC-704865) as Second-Line Therapy for Patients with Metastatic Colorectal Cancer who have Progressed on Bevacizumab with Either FOLFOX, OPTIMOX or XELOX | Both | Included | 72 | Relapsed |
| S0622 | Phase II Studies of Two Different Schedules of Dasatinib (NSC-732517) in Bone-Metastasis Predominant Metastatic Breast Cancer | Both | Included | 85 | Newly diagnosed/ Relapsed |
| S0635 | A Phase II Trial of the Combination of OSI-774 (Erlotinib; NSC-718781) and Bevacizumab (rhuMAb VEGF; NSC-704865) in Stage IIIB and IV Bronchioloalveolar Carcinoma (BAC) and Adenocarcinoma with BAC Features (AdenoBAC) | Both | Included | 84 | Newly diagnosed/ Relapsed |
| S0636 | A Phase II Trial of the Combination of OSI-774 (Erlotinib; NSC-718781) and Bevacizumab (rhuMAb VEGF; NSC-704865) in Never-Smokers with Stage IIIB and IV Primary NSCLC Adenocarcinomas | Both | Included | 89 | Newly diagnosed |
